# Acute pseudo-obstruction of the large bowel with caecal perforation following normal vaginal delivery: a case report

**DOI:** 10.1186/1752-1947-4-123

**Published:** 2010-04-29

**Authors:** David Cartlidge, Marlon Seenath

**Affiliations:** 1 Department of Surgery, University Hospital of North Staffordshire, Newcastle-under-Lyme, Stoke-on-Trent, ST4 6QG, UK

## Abstract

**Introduction:**

Acute pseudo-obstruction of the large bowel following normal vaginal delivery is an extremely rare complication of normal vaginal delivery. It can be fatal if not recognized early. Only one previous report has been found in the English literature.

**Case presentation:**

A 36-year old Caucasian, normally fit woman presented with abdominal distension and vomiting five days post-normal vaginal delivery at term. Localised peritonitis in the right iliac fossa developed in the next few days, and caecal perforation was found at laparotomy, without evidence of appendicitis or colitis.

**Conclusion:**

Although very rare, Ogilvie's syndrome should be considered by obstetricians, general surgeons and general practitioners as a potential cause of vomiting and abdominal pain following normal vaginal delivery. Early recognition and management are essential to minimize the possibility of developing serious complications.

## Introduction

Ogilvie's syndrome is a rare but potentially life-threatening obstetric complication consisting of an acute pseudo-obstruction of the large bowel [1]. The most important potential complication of the condition is large bowel perforation with subsequent fecal peritonitis and associated high mortality [2]. Several cases of colonic perforation secondary to Ogilvie's syndrome have been reported following Caesarian section, but only one report in the English literature was found at the time of writing that has described the condition post-normal vaginal delivery [3].

## Case presentation

A 36-year-old Caucasian woman was admitted to our hospital with abdominal distension, pain and vomiting five days following the normal vaginal delivery of her second child born at term. Our patient was normally fit and well with a medical history of recurrent urinary tract infections and mild asthma. Folic acid tablets were the only medications that our patient had been taking during the course of her pregnancy, and there was no history of constipation. Her vaginal delivery had been uncomplicated except for a third degree tear during delivery, which requires suturing in theatre. Our patient had been commenced on laxatives following the repair of the perineal tear, and showed no symptoms of perineal sepsis.

**Figure 1:**
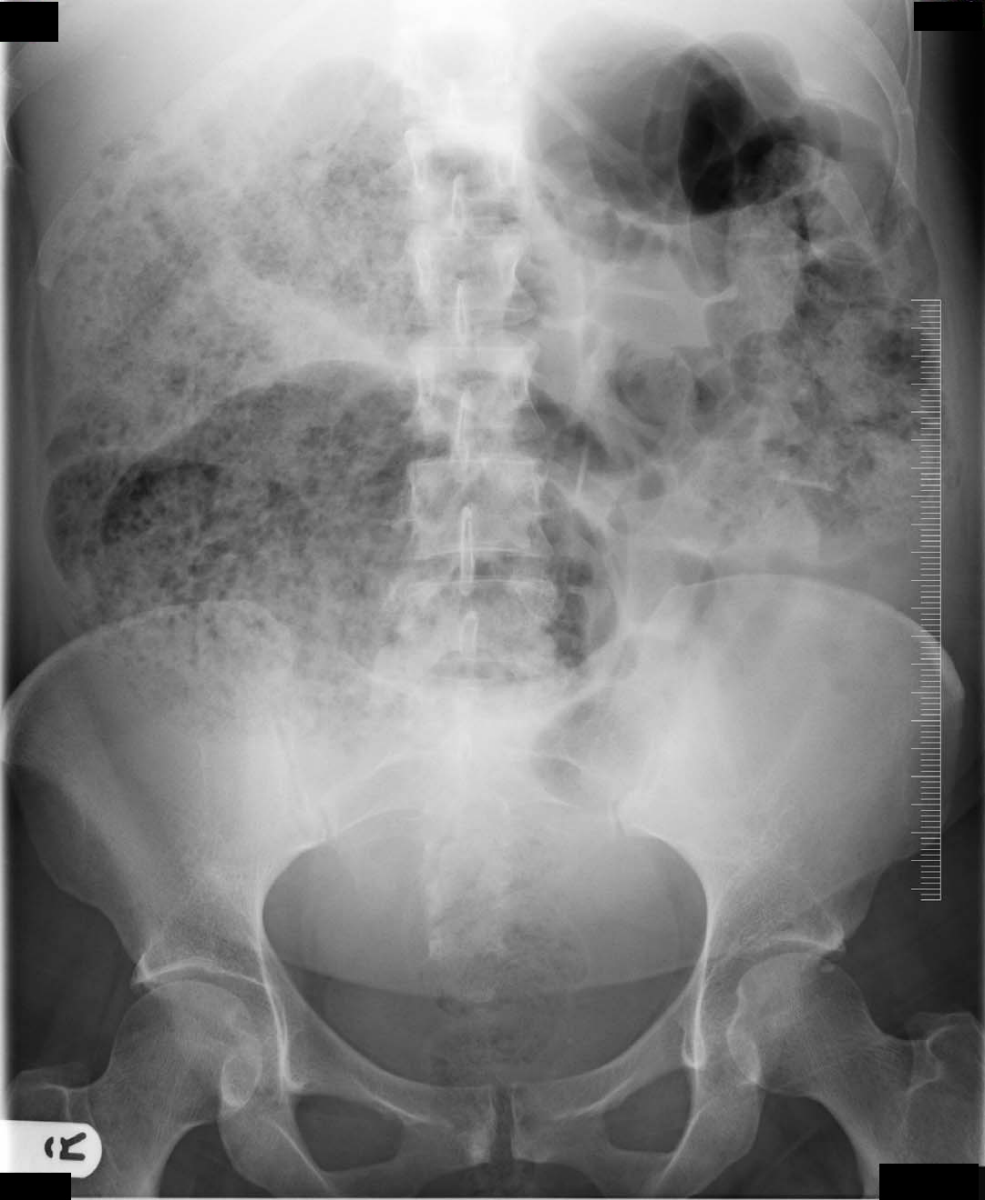
**Abdominal radiograph**.

**Figure 2:**
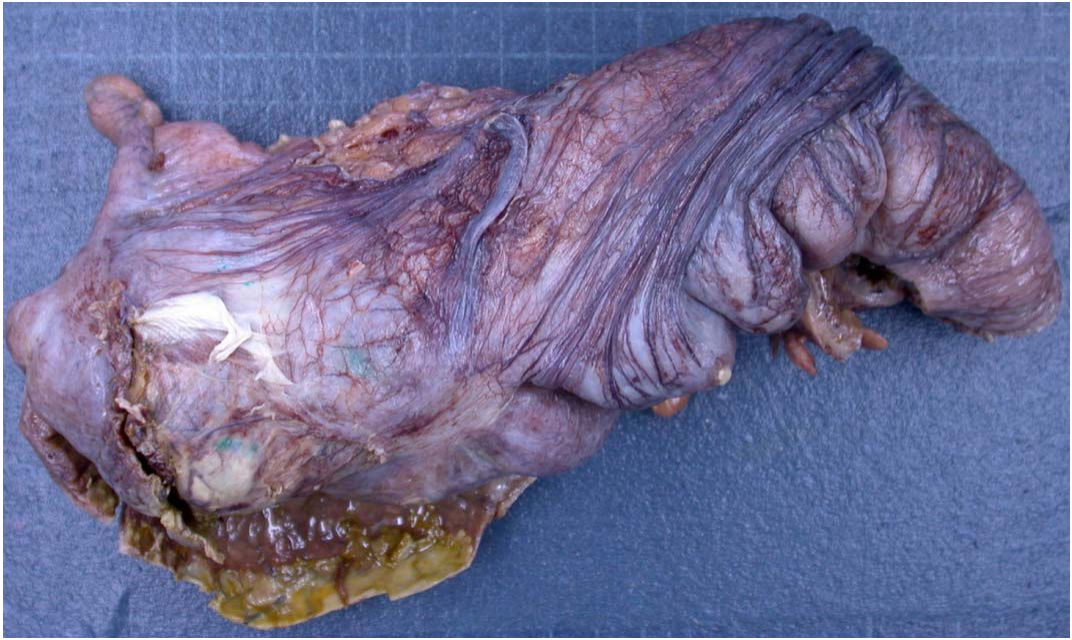
**Gross specimen of ascending colon with perforation**.

**Figure 3:**
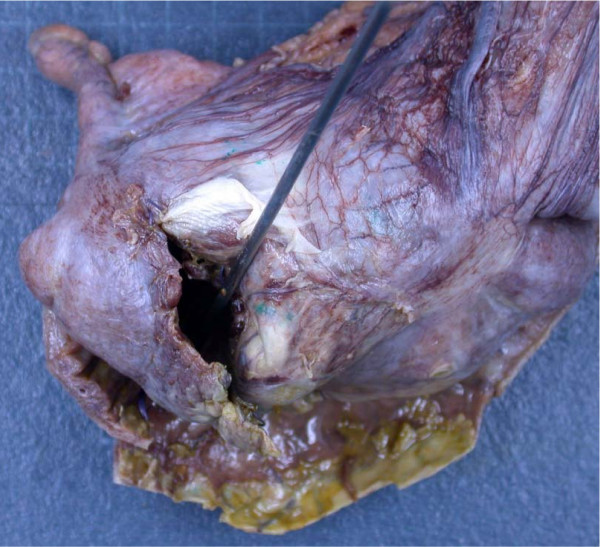
**Perforated ascending colon with surrounding exudate**.

On examination, during the acute admission five days after the normal vaginal delivery, our patient was found to have a tympanitic, distended abdomen. Our patient had opened her bowels on one occasion since giving birth. She was noted to have a leukocytosis and elevated C-reactive protein (12.2 × 10^9^/L and 322.0 mg/L respectively). Serum sodium and potassium were within normal limits. Her symptoms worsened over the next three days with increased vomiting and signs of a localized peritonitis in the right iliac fossa, absent bowel sounds and pyrexia. Abdominal X-ray showed a grossly dilated proximal colon and our patient was taken to theatre for exploratory laparotomy (Figure 1). At laparotomy, a 4 × 4 cm perforation in the cecum was found with no evidence of acute appendicitis or colitis. A limited right hemicolectomy was performed (Figures 2 and 3) with a stapled side to side ileocolic anastomosis. She made an uneventful recovery and was discharged home on day seven. Histological examination of the right colon revealed ischemic changes.

## Discussion

The exact etiology of Ogilvie's syndrome is unknown, but it has been associated with several disease processes such as trauma, abdominal and/or pelvic surgery, and sepsis [1]. Bed rest and abnormal electrolytes are also listed as factors associated with the development of the syndrome [4], and Strecker *et al. *reported that the association between Ogilvie's syndrome and vaginal delivery may be due to the declining serum oestrogen levels in the post-partum period [5]. In the only other reported case of Ogilvie's syndrome following normal vaginal delivery in the English literature, the histological findings of the caecum after right hemicolectomy showed no specific pathology [3].

The mechanism of the condition is thought to involve loss of tone in the parasympathetic nerves S2 to S4. This, in turn, results in an atonic distal colon and pseudo-obstruction [6]. Various sources report a cut-off sign relating to an area of dilated and collapsed bowel around the splenic flexure corresponding to the transition zone between the vagal and sacral parasympathetic nerve supplies [4,7]. The cut-off sign is used to support the hypothesis of parasympathetic inhibition causing Ogilvie's syndrome [4,7].

The diagnosis of Ogilvie's syndrome is widely reported to be troublesome due to the non-specific clinical features [3]. Abdominal distension is considered to be the common symptom, and Jetmore *et al. *report no known cases of Ogilvie's syndrome to have presented without distension of the abdomen [4]. As with any case of suspected ileus or obstruction, electrolyte levels are an essential investigation and in the 48 cases of Ogilvie's syndrome reported by Jetmore *et al.*, 83% demonstrated at least one electrolyte disturbance with hypocalcaemia being the most common [4]. Abdominal radiography is a standard first-line investigation, and Keswani *et al. *reported that a caecal diameter of nine cm or more is the 'only definitive sign of imminent perforation' [3].

Several sources have discussed non-surgical management options with decompression of the bowel with intravenous fluid support as the standard of treatment. Jetmore *et al. *advocate for colonoscopic decompression as a successful method of avoiding surgical management, unless signs of peritonism are evident [4]. Stephenson *et al. *have shown that the use of prokinetic, parasympathomimetic drugs such as neostigmine can be successful in the management of Ogilvie's syndrome, although the benefit in cases of idiopathic Ogilvie's syndrome is not certain [7]. With regard to post-partum patients, Strecker *et al. *support the use of laxatives in the post-partum period and stress the importance of early diagnosis [5].

## Conclusion

Ogilvie's syndrome following normal vaginal delivery in pregnant women is an extremely rare but serious condition requiring early recognition and treatment to prevent potentially fatal complications. General practitioners, obstetricians and surgeons must be aware of the non-specific presenting features and exercise a high index of suspicion of otherwise unexplained abdominal distension in the post-partum period.

## Competing interests

The authors declare that they have no competing interests.

## Authors' contributions

DC and MS identified the case and gathered research in the form of literature reviews. DC gathered the investigation results including the figures used in the manuscript. DC wrote the final report, which was read and approved by MS.

## Consent

Written consent was obtained from our patient for publication of this case report and any accompanying images. A copy of the written consent is available for review by the Editor-in-Chief of this journal.
